# Automatic determination of cardiovascular risk by CT attenuation correction maps in Rb-82 PET/CT

**DOI:** 10.1007/s12350-017-0866-3

**Published:** 2017-04-04

**Authors:** Ivana Išgum, Bob D. de Vos, Jelmer M. Wolterink, Damini Dey, Daniel S. Berman, Mathieu Rubeaux, Tim Leiner, Piotr J. Slomka

**Affiliations:** 1 Image Sciences Institute, University Medical Center Utrecht, Heidelberglaan 100, 3584 CX Utrecht, The Netherlands; 2 Departments of Imaging and Medicine, Cedars-Sinai Medical Center, 8700 Beverly Blvd Ste. A047N, Los Angeles, CA 90048 USA; 3 Department of Radiology, University Medical Center Utrecht, Heidelberglaan 100, 3584 CX Utrecht, The Netherlands

**Keywords:** Automatic calcium scoring, coronary calcium, CT attenuation correction map, cardiac CT, cardiovascular risk

## Abstract

**Background:**

We investigated fully automatic coronary artery calcium (CAC) scoring and cardiovascular disease (CVD) risk categorization from CT attenuation correction (CTAC) acquired at rest and stress during cardiac PET/CT and compared it with manual annotations in CTAC and with dedicated calcium scoring CT (CSCT).

**Methods and Results:**

We included 133 consecutive patients undergoing myocardial perfusion ^82^Rb PET/CT with the acquisition of low-dose CTAC at rest and stress. Additionally, a dedicated CSCT was performed for all patients. Manual CAC annotations in CTAC and CSCT provided the reference standard. In CTAC, CAC was scored automatically using a previously developed machine learning algorithm. Patients were assigned to a CVD risk category based on their Agatston score (0, 1-10, 11-100, 101-400, >400). Agreement in CVD risk categorization between manual and automatic scoring in CTAC at rest and stress resulted in Cohen’s linearly weighted *κ* of 0.85 and 0.89, respectively. The agreement between CSCT and CTAC at rest resulted in *κ* of 0.82 and 0.74, using manual and automatic scoring, respectively. For CTAC at stress, these were 0.79 and 0.70, respectively.

**Conclusion:**

Automatic CAC scoring from CTAC PET/CT may allow routine CVD risk assessment from the CTAC component of PET/CT without any additional radiation dose or scan time.

**Electronic supplementary material:**

The online version of this article (doi:10.1007/s12350-017-0866-3) contains supplementary material, which is available to authorized users.

## Introduction

Positron emission tomography (PET) myocardial perfusion imaging (MPI) is a well-established noninvasive procedure for evaluation of hemodynamically significant coronary artery disease (CAD). However, PET is not able to visualize atherosclerotic plaque, and hence, it does not allow detection of subclinical CAD.^1^ The coronary artery calcium (CAC) burden determined in calcium scoring CT (CSCT), quantified as the CAC score, is a strong and independent predictor of cardiovascular events (CVE)^2^ and adds prognostic and diagnostic information to an MPI scan.^1^ Previous studies suggest that CT attenuation correction (CTAC) images that are acquired as a part of PET/CT or SPECT/CT may potentially enable the identification and quantification of atherosclerotic calcifications in the coronary arteries, and thereby allow improved assessment of CVE risk.^3^,^4^ If the CTAC component of the cardiac MPI could be used for calcium scoring and thereby for the improved evaluation of CVE risk and improved diagnosis,^1^ the acquisition of a dedicated CSCT scan could be omitted, reducing ionizing radiation dose for the patients, shortening the study time, and simplifying the procedure. However, CTAC images are not acquired using an optimized protocol for calcium scoring. Excessive motion artifacts, low image resolution, and high levels of image noise make manual calcium scoring in CTAC a tedious task. Hence, an automatic method would be advantageous in clinical routine. In this work, we investigate the utility of a novel method for fully automatic calcium scoring applied to CTAC images. The method is specifically optimized to score CAC in rest and stress CTAC images that are acquired as part of cardiac PET/CT MPI exams. Additionally, we compare cardiovascular (CVD) risk categorization obtained from dedicated CSCT scans with manual and automatic CAC scoring in CTAC images.

## Methods

### Patients

The study included consecutive 133 patients (85 men, 48 women, average age 69, age range 31-97) undergoing ^82^Rb PET/CT and dedicated CSCT scanning at Cedars-Sinai Medical Center. Table 1 lists patients’ demographic and clinical data. All patients provided written informed consent for the use of their clinical and imaging data for research purposes, and the study was approved by the Institutional Review Board.

**Table 1:** Demographics and clinical characteristics of the patients

Patient data	
Age (years)	69 ± 12
Male	85 (64%)
Body mass index (kg/m^2^)	28 ± 6
Diabetes	50 (38%)
Hypertension	100 (75%)
Hypercholesterolemia	78 (59%)
Smoking	20 (15%)

### Imaging Protocols

#### PET

All patients underwent rest and stress gated ^82^Rb PET MPI and CSCT imaging on a hybrid Biograph 64 PET/CT scanner.^5^ A 6-minute rest acquisition started immediately before the injection of 925-1850 MBq (25-50 mCi) of ^82^Rb. Pharmacological stress was induced using intravenous adenosine infusion^6^ or using a regadenoson bolus.^7^ No adjunctive exercise was performed. A 6-minute stress acquisition started immediately with the injection of 925-1850 MBq (25-50 mCi) of ^82^Rb.

#### CTAC

Separate CTAC scans were acquired immediately after stress and rest emission PET studies, without moving the patient from the scan table. The CTAC parameters were 100 kVp, pitch 1.5, 11 mAs, duration 3.4 seconds, 3 mm slice thickness, end-expiration breath hold.

#### CSCT

After completion of PET MPI, without moving patients from the scan table, CSCT scan was acquired. CSCT scans (120 kVp, 11 mAs, 3 mm slice thickness and increment, ECG-triggering) consisted of 30 to 40 slices encompassing the heart from the carina to the apex, with a 30-35 cm field of view sufficient to include the entire heart as well as the ascending and descending thoracic aortas.

### Manual Calcium Scoring

In CSCT scans, CAC scoring was performed as part of clinical routine using dedicated CT vendor software (SciImage, California US). This software applies a standard 130 Hounsfield unit (HU) threshold for calcification extraction. For each patient, the Agatston score was computed. Subjects were assigned to a CVD risk category based on the Agatston score (0, 1-10, 11-100, 101-400, >400).^8^,^9^

In CTAC scans, CAC scoring was performed by an experienced observer using dedicated in-house developed software^9^ using a standard 130 HU threshold. To minimize the effect of intraobserver variability, CTAC scans of each patient acquired at rest and stress were scored consecutively. The observer was blinded to CAC annotations in CSCT scans. In CTAC scans, number of lesions, Agatston, and volume calcium scores were computed.^10^

### Automatic Calcium Scoring from Attenuation Maps

Coronary calcifications were identified using our previously developed algorithm for automatic CAC scoring from CSCT.^9^,^11^ The algorithm was originally developed for scans obtained with settings optimized for calcium quantification, including ECG triggering minimizing the effect of cardiac motion. Given that CTAC scans are obtained for attenuation correction and are made using suboptimal image acquisition settings for calcium scoring, scans are affected by substantial image noise, artifacts caused by cardiac motion due to lack of ECG synchronization, and partial volume effect caused by the large in-plane resolution. Hence, for this work, the algorithm was specifically optimized for CTAC images.

The algorithm first identifies potential CAC lesions in the image by intensity-based thresholding and 3D connected-component labeling, thus following the standard clinical procedure. Single voxel lesions are removed from further analysis as they cannot be differentiated from the noise. Subsequently, each potential calcified lesion is characterized by a number of features, such as volume, shape, intensity and location. Shape features are calculated as ratios of eigenvalues found using a principal component analysis on the location of the voxels within each candidate. Intensity features are computed using the intensity value statistics of each lesion and Gaussian filters at different resolutions. Location features describe the spatial position of each candidate using an estimate of the location of the coronary artery tree, which is acquired using multi-atlas based segmentation. Once features for all potential CAC lesions have been computed, a two-class classifier separates CAC and negative lesions such as calcifications in the aorta, bones, or noise. The classifier consists of an ensemble of 250 extremely randomized decision trees (Extra-Trees).^9^,^12^

Tenfold cross-validation experiments were performed by dividing the CTAC data into training sets containing 90% of the images and test sets containing the remaining 10% of images. Tenfold cross-validation allows strict per patient separation of training and test data and maximizes the amount of training data. For each candidate lesion, the algorithm determined the probability that it was CAC and labeled it as either CAC or background. The identified CAC was quantified using the same procedure as in manual scoring.

### Validation

To evaluate whether automatic CAC scoring applied to CTAC images is feasible, the CAC scores obtained manually and automatically were compared. This analysis was performed separately for CTAC images acquired during rest and stress.

To evaluate whether CAC scoring in CSCT images could be substituted by CAC scoring in CTAC scans, manual and automatic CAC scores determined in CTAC images were compared with CAC scores determined in CSCT scans.

### Statistical Analysis

To evaluate the performance of automatic CAC scoring in CTAC scans, per patient sensitivity and a number of false positives using the number of lesions and their volume were determined. The reliability of the Agatston scores was determined by the two-way mixed intraclass correlation coefficient (ICC) for absolute agreement between manual and automatic scores in CTAC images. In addition, we compared CAC scores in CSCT with manual and automatic CAC scores in CTAC images. The analyses were performed separately for CTAC scans acquired at rest and stress. Furthermore, we evaluated whether there was a difference between Agatston scores derived from CTAC images acquired at rest and at stress, with either manual or automatic scoring, by Wilcoxon signed-rank test. Bonferroni correction was applied to correct for multiple pairwise comparisons, so that the level of significance was set to 0.0083.

To evaluate accuracy and agreement of CVD risk categorization between automatic and manual CAC scoring in CTAC scans, patients were assigned to one of the standard five CVD risk categories (0, 1-10, 11-100, 101-400, >400).^8^ It is likely that for patients scanned with two different CT settings, obtained CAC scores may differ but the ranking of the patient scores remains the same. Therefore, we compared CVD risk categorization between CTAC and CSCT images by first determining the number of patients assigned to each CVD risk group according to the Agatston scores in CSCT scans. Subsequently, the Agatston scores obtained in CTAC scans were ranked, and we assigned the same number of patients to each group as determined by their ranking with respect to the CSCT categorization.^13^ The analysis was performed separately for CTAC scans at rest and stress, and separately for manual and automatic scoring in these images.

The accuracy of CVD risk categorization was determined as a percentage of scans in which two methods agree. The proportion of agreement beyond chance between CVD risk category assignments was determined using Cohen’s linearly weighted κ. Comparisons between kappa values and accuracies were performed by Z-score test. Statistical analysis was performed using IBM SPSS Statistics (Version 23. Armonk, NY: IBM Corp.). Cohen’s *κ* was computed using VassarStats analysis (http://vassarstats.net/kappa.html).

## Results

### Automatic vs Manual Calcium Scoring in CTAC Scans

Five patients were excluded due to high levels of image noise^4^ and artifacts caused by metal implants^1^ preventing manual CAC scoring.

In rest CTAC scans, the algorithm correctly detected 68.5% CAC lesions and 76.4% CAC volume per scan, with on average 0.3 false positive lesions corresponding to 25 mm^3^ false positive volume per scan. The two-way ICC for absolute agreement between the automatically determined and reference (manual) Agatston scores was 0.828 (CI 0.774-0.882).

The comparison of CVD risk categorization between expert and automatic CAC scoring are listed in Table 2a. The method automatically assigned the reference risk category to 106/128 (82.8%) patients. Linearly weighted *κ* was 0.85 (CI 0.78-0.91) indicating excellent agreement.

**Table 2:** Manual vs automatic CAC scoring in CTAC at (a) rest and (b) stress

	0	1–10	11–100	101–400	>400
0	17	0	0	0	0
1–10	3	2	1	0	0
11–100	2	0	14	1	1
101–400	2	0	4	30	1
>400	0	0	0	7	43

	0	1–10	11–100	101–400	>400
0	18	0	2	0	0
1–10	0	2	0	0	0
11–100	1	1	22	0	0
101–400	1	0	3	28	2
>400	0	0	0	5	43

Cardiovascular risk categories based on the Agatston score (0, 1-10, 11-100,101-400, >400) assigned to a patient by the reference manual scoring (rows) and automatic algorithm (columns) in CTAC scans acquired at (a) rest and (b) stress

In stress CTAC scans, on average the algorithm correctly detected of 75.7% CAC lesions and 83.5% CAC volume per scan, with on average 0.3 false positive lesions corresponding to 23 mm^3^ false positive volume per scan.

**Figure 1:**
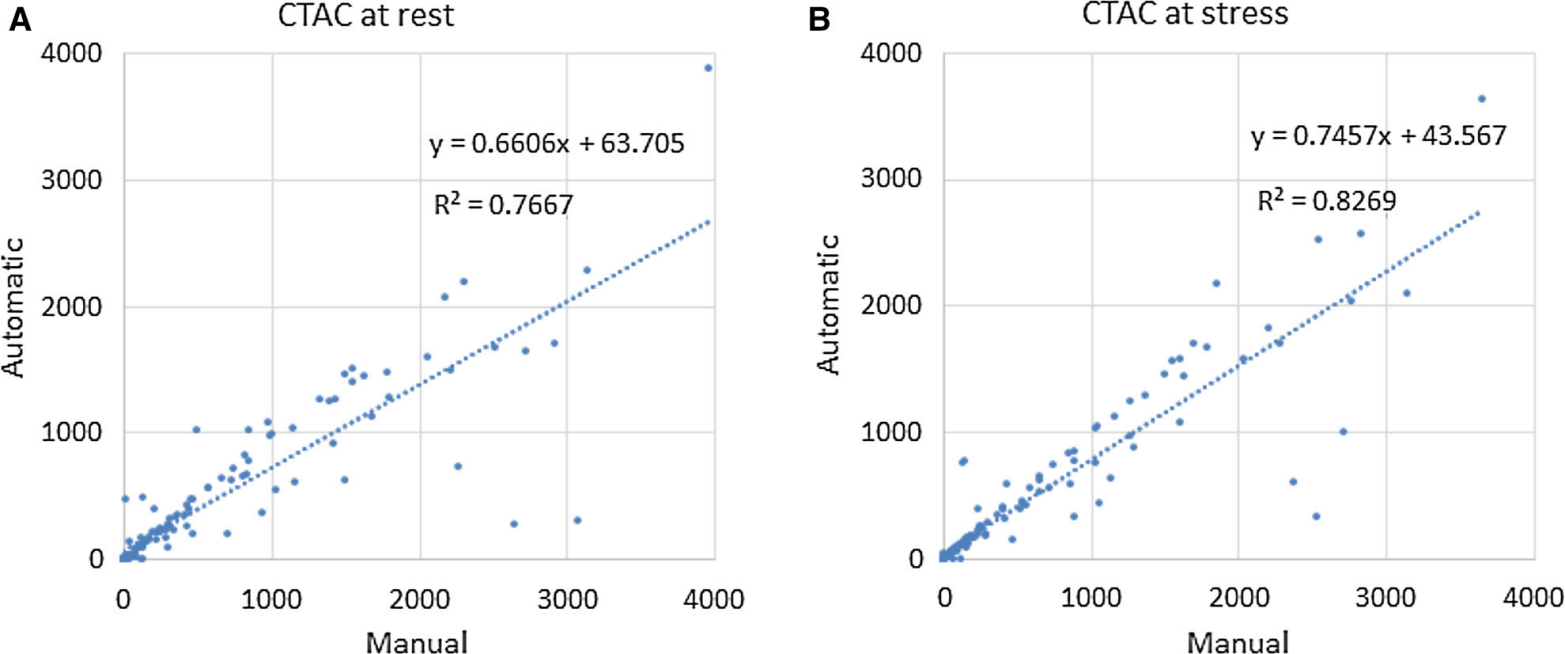
Manually determined (*x*-axis) vs automatically computed (*y*-axis) CAC Agatston scores in **A** CTAC images at rest and **B** CTAC images at stress

The two-way ICC between the automatically determined and reference Agatston scores was 0.884 (CI 0.829-0.920). Results of the CVD risk category assignment are listed in Table 2b. The method automatically assigned the reference risk category to 113/128 (88.3%) patients. Linearly weighted *κ* was 0.89 (CI 0.84-0.95) indicating excellent agreement. Figure 1 shows manually vs automatically determined CAC scores in CTAC images at rest and stress.

**Figure 2:**
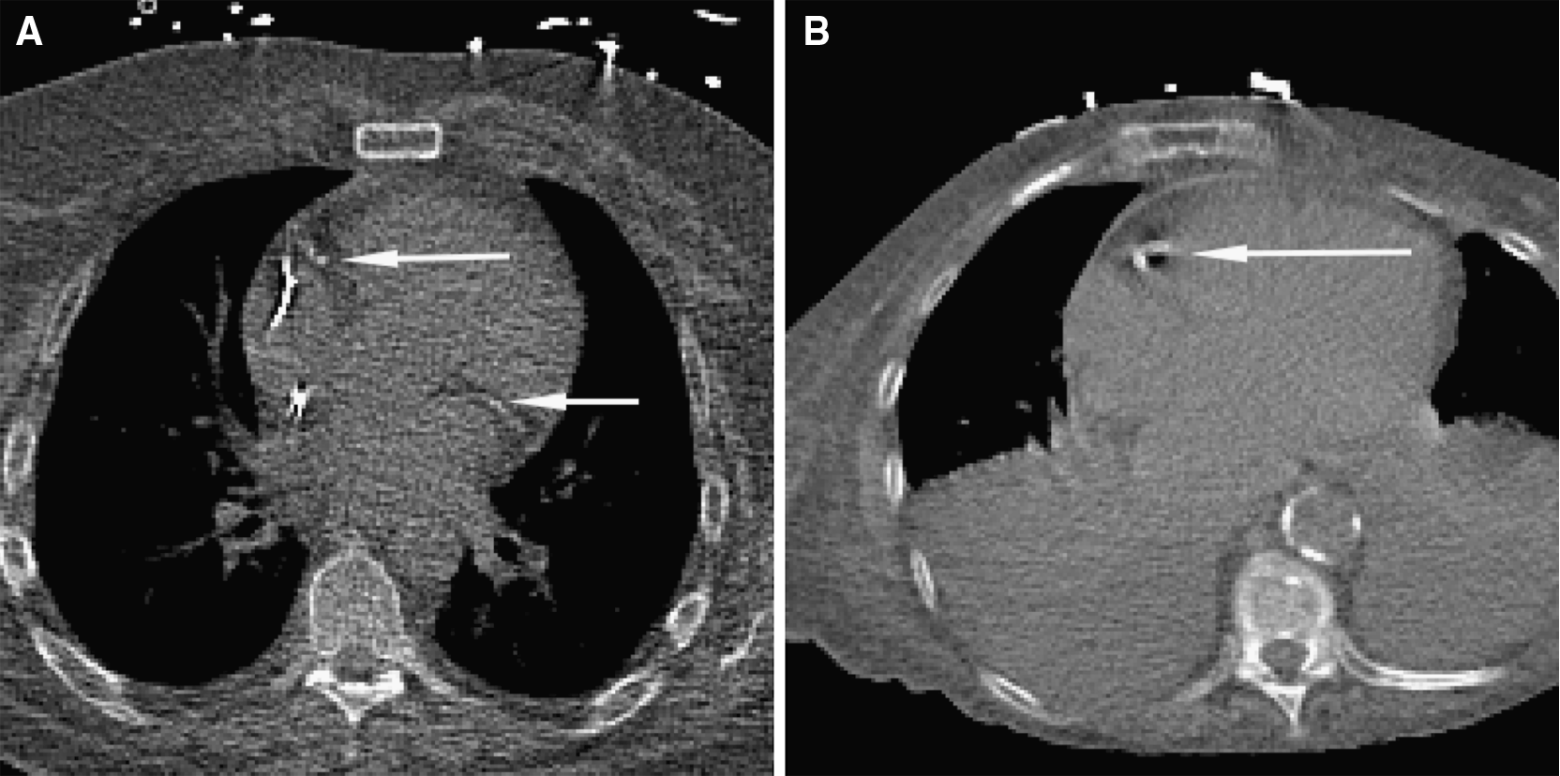
Examples of calcifications correctly detected by the automatic algorithm in CTAC scans. **A** CAC lesions in the RCA and LCX in CTAC at rest in a scan with metal implants. **B** CAC in the RCA strongly affected by cardiac motion in CTAC scan at stress showing severe abnormalities in the lungs

**Figure 3:**
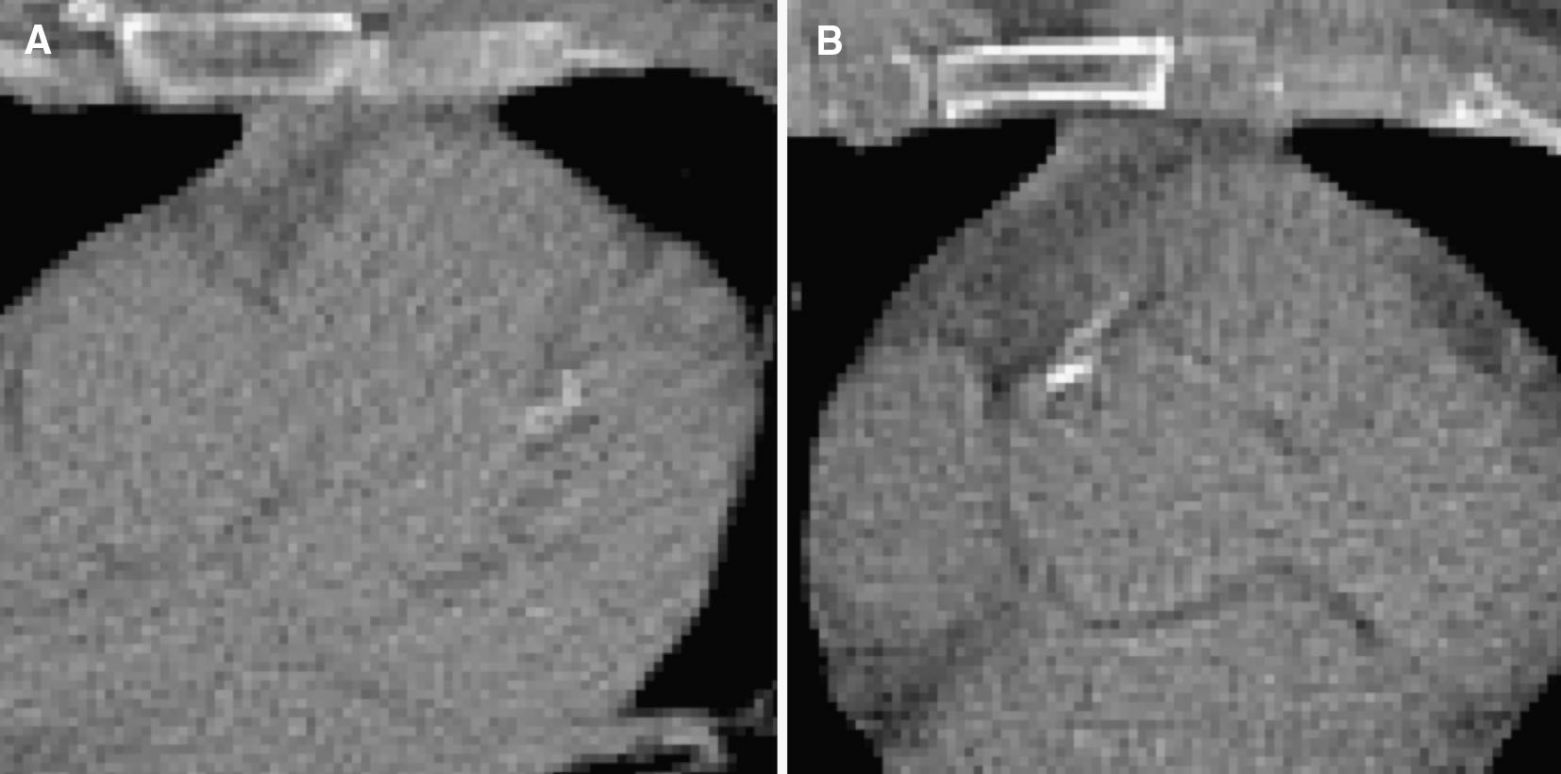
**A** CAC in LAD missed by the automatic algorithm that resulted in underestimation of CVD risk. The CAC lesion appears blurred, probably due to cardiac motion and large pixel size. **B** Calcification in the ascending aorta near the right coronary ostium detected as CAC by the automatic method. This large false positive lesion caused overestimation of CVD risk categorization

The results demonstrate that the automatic method can detect calcifications in CTAC scans in the presence of metal implants and in scans that are strongly affected by cardiac motion (Figure 2). Nevertheless, inspection of the automatic results in CTAC scans revealed several outliers. The automatic method occasionally missed large CAC lesions affected by cardiac motion or partial volume effect resulting in low contrast with the surrounding tissue (Figure 3a). This led to underestimation of the CVD risk. Similarly, the automatic method occasionally incorrectly detected noise voxels or calcifications in the aorta at the coronary ostia, leading to overestimation of the CVD risk (Figure 3b). False negative lesions were often larger than false positives, leading to more frequent under- than overestimation of the CAC burden and consequently of the CVD risk category (14% vs 3% at rest; 9% vs 3% at stress). False negative and false positive lesions were similar in CTAC images at rest and stress. Observed errors of the automatic method are in agreement with the results reported by Wolterink et al.^9^ in the application of the same method to CSCT data.

### Manual and Automatic Calcium Scoring in CTAC vs CSCT Scans

**Figure 4:**
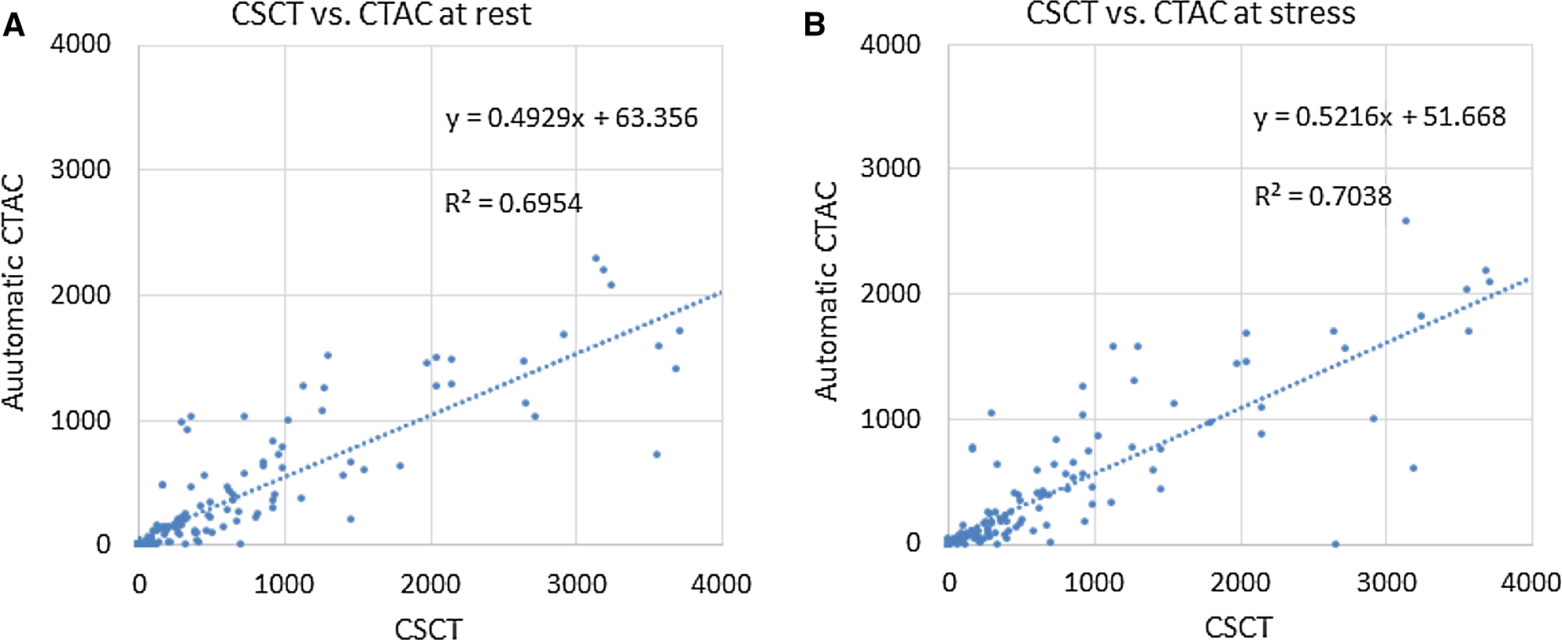
Manually determined CAC Agatston scores in CSCT (*x*-axis) vs automatically computed CAC scores in CTAC (*y*-axis) **A** at rest and **B** at stress

The two-way ICC for absolute agreement between manual expert Agatston scores in the dedicated CSCT scans and the CTAC are 0.863 (CI 0.767-0.915) for manual scores, and 0.678 (CI 0.455-0.800) for automatic scores at rest, and 0.866 (CI 0.738-0.924) for manual scores, and 0.702 (CI 0.489-0.817) for automatic scores in stress CTAC scans. Figure 4 shows CAC scores determined manually in references CSCT images vs automatically obtained CAC scores in CTAC at rest and stress. There was a significant difference between Agatston scores in CSCT and Agatston scores in CTAC (*P* ≪ .01). This was the case for CTAC scans acquired at rest and stress as well as for automatic and manual CAC scoring. However, there was no significant difference between manual Agatston scores in CTAC at rest and CTAC at stress (*P* = .678), nor between automatic Agatston scores in CTAC at rest and CTAC at stress (*P* = .281).

Agreements in CVD risk categorization between manual expert scoring in dedicated CSCT scans with manual and automatic scoring in CTAC scans during rest and during stress are shown in Tables 3 and 4. The corresponding accuracies and linearly weighted *κ* values are listed in Table 5, indicating good to excellent agreement of both manual and automatic scoring.

**Table 3:** Manual CAC scoring in CSCT vs (a) manual and (b) automatic CAC scoring in CTAC at rest

	Very low	Low	Intermediate	High	Very high
Very low 0	21	1	0	0	0
Low 1–10	5	0	1	0	0
Intermediate 11–100	0	2	6	4	0
High 101–400	0	0	3	25	7
Very high >400	0	0	1	6	55

	Very low	Low	Intermediate	High	Very high
Very low 0	13	0	0	0	0
Low 1–10	5	0	1	0	0
Intermediate 11–100	4	0	3	5	0
High 101–400	2	0	2	22	9
Very high >400	0	0	1	8	53

Cardiovascular risk categories based on the Agatston score (0, 1-10, 11-100, 101-400, >400) assigned to a patient by the manual scoring in CSCT (rows) and (a) manual and (b) automatic scoring in CTAC scans acquired at rest (columns) taking different ranges of Agatston scores between CSCT and CTAC scan into account

**Table 4:** Manual CAC scoring in CSCT vs (a) manual and (b) automatic CAC scoring in CTAC at stress

	Very low	Low	Intermediate	High	Very high
Very low 0	13	0	0	0	0
Low 1–10	5	0	1	0	0
Intermediate 11–100	1	0	6	5	0
High 101–400	0	0	3	24	8
Very high >400	1	0	1	6	54

	Very low	Low	Intermediate	High	Very high
Very low 0	11	0	1	1	0
Low 1–10	5	0	1	0	0
Intermediate 11–100	1	0	6	5	0
High 101–400	2	0	3	20	10
Very high >400	1	0	2	7	52

Cardiovascular risk categories based on the Agatston score (0, 1-10, 11-100, 101-400, >400) assigned to a patient by the manual scoring in CSCT (rows) and (a) manual and (b) automatic scoring in CTAC scans acquired at stress (columns) taking different ranges of Agatston scores between CSCT and CTAC scan into account

**Table 5:** CAC scoring in CSCT vs. manual and automatic CAC scoring in CTAC

	CSCT vs CTAC rest		CSCT vs CTAC stress	
	Manual	Automatic	Manual	Automatic
Accuracy [CI]	0.76 [0.68–0.82]	0.71 [0.62–0.78]	0.77 [0.69–0.83]	0.70 [0.62–0.77]
*κ* [CI]	0.82 [0.76–0.88]	0.74 [0.65–0.82]	0.79 [0.73–0.89]	0.70 [0.61–0.80]

Accuracy (top) and linearly weighted Cohen’s *κ* for CVD risk category assignment (bottom) with corresponding 0.95% confidence interval between manual expert calcium scoring in dedicated CSCT, and manual and automatic calcium scoring in CTAC images acquired at rest and stress taking different ranges of Agatston scores between CSCT and CTAC scan into account. There were no significant differences between *κ* values and accuracies for stress vs rest and for for automatic vs manual scoring

**Figure 5:**
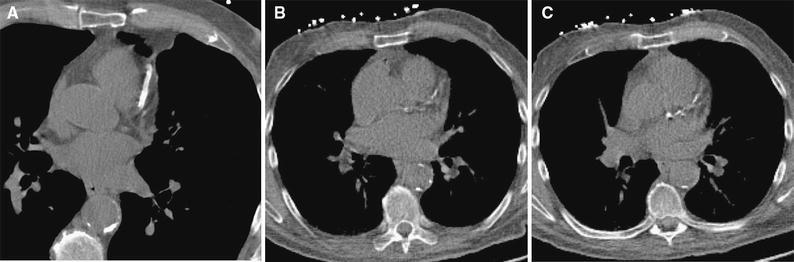
One slice from **A** CSCT, **B** CTAC at rest and **C** CTAC at stress of the same patient showing the LAD. The CSCT scan clearly visualizes a coronary artery stent, while the same stent appears as somewhat elongated CAC in the LAD causing large overestimation of the CAC score in CTAC images

**Figure 6:**
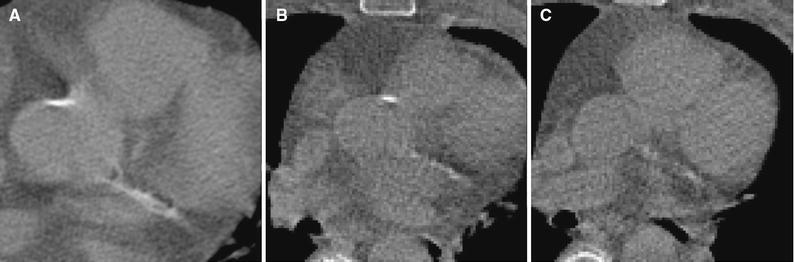
One slice from **A** CSCT, **B** CTAC at rest and **C** CTAC at stress of the same patient showing a large CAC in the LCX. While CAC in the CSCT appears large, only some of its voxels exceed 130 HU threshold value leading to substantial CVD risk underestimation (Agatston scores 163, 11, and 6, respectively)

Even though results demonstrate that manual expert CAC scoring in CTAC tends to slightly better agree with expert scoring in CSCT than automatic CAC scoring in CTAC scans, visual inspection of the images suggests that differences in image characteristics between CSCT and CTAC scans have a stronger influence on the CAC scores than false positive and false negative errors made by the automatic algorithm. Figures 5 and 6 illustrate differences between the image characteristics and their impact on the visualization of CAC lesions.

## Discussion

This study evaluated both manual and automatic coronary calcium scoring in CTAC images acquired during PET/CT scanning. In addition, the obtained CAC scores in CTAC images were compared with manual expert CAC scores in CSCT scans to evaluate the potential of CAC scoring in CTAC images and thereby assess the possibility of using CTAC maps alone for cardiac risk assessment. A comparison of manual and automatic CAC scoring in CTAC images showed that the vast majority (>83%) of patients could be assigned to the correct CVD risk category based on automatic risk assessment.

Compared with manual CAC scores in CTAC images, automatic scoring in images at stress tends to result in slightly better performance than automatic scoring in images at rest (ICC: 0.884 and 0.828, and *κ* 0.89 and 0.85 in stress and rest, respectively). Visual inspection of the images did not reveal obvious causes. However, it appeared that errors were similar in both scans suggesting that combining the automatic results from both images would not lead to a better agreement between the scores in CTAC and CSCT scans. Note that there was no statistically significant difference between the manual CAC scores in CTAC at rest and at stress, nor between the automatic CAC scores in CTAC at rest and stress.

To the best of our knowledge, very few studies have investigated quantitative CAC scoring from standard CTAC scans obtained in PET or SPECT^3^,^4^,^14^. In a multicenter study including 492 patients, Einstein et al.^3^ compared visual assessment of CVD risk from low-dose CTAC with CVD risk categorization using quantitative Agatston scoring in CSCT. The analysis resulted in 63% accuracy and linearly weighted *κ* of 0.89. As compared to our study, we observed similar results 70%-77% accuracy and linearly weighted *κ* of 0.70 to 0.82. However, we used five risk categories in our study (without >1000 category), and therefore, the results cannot be directly compared. In comparison with a study by Einstein et al., we evaluated the CAC scoring separately for rest and stress CTAC scans and also evaluated the performance of the automated method. In two other recent studies, Mylonas et al.^4^ and Kaster et al.^14^ scored calcium using the manual identification of coronary calcifications, while we evaluated fully automatic quantification in CTAC scans. On a set of 91 patients, Mylonas et al.^4^ reported an ICC of 0.804 between manually obtained Agatston scores in CSCT and CTAC during rest using a 130 HU threshold for CAC scoring. In the current study, in a set of 128 patients, this agreement was 0.863 and 0.866 for rest and stress, respectively. This difference might have been caused by several factors. First, in our study, CTAC and CSCT scans were made on the same day, without moving the patient off the table, while in Mylonas et al.^4^ the scans were acquired within 6 months’ time interval. Even though changes in calcium burden within this period are unlikely, they might have occurred. However, it is more likely that interscan variability, additionally caused by different patient positioning on the table, might have had a larger influence on the CAC scores. Second, Mylonas et al.^4^ evaluated agreement between manual scoring in CTAC images vs CSCT using different threshold levels and obtained best results using an intensity value threshold of 50 HU with ICC of 0.953. Moreover, Kaster et al.^14^ reported the best agreement using an 110 HU threshold. However, in the set of images used in our study, CAC scoring using an 110 and 50 HU threshold level did not allow separation of calcifications from the background. Using such low thresholds frequently connected CAC lesions with bony structures. The standard intensity value threshold of 130 HU seemed to agree best with the visual assessment of coronary artery calcifications in our images. Nevertheless, dedicated investigation on determining the best threshold level for coronary calcium scoring in CTAC scans might further improve the agreement with calcium scores in CSCT images, but it is beyond the scope of this study.

In literature, CVD risk categories have been defined in various ways. In dedicated CSCT scans, five (Agatston scores 0, 1-10, 11-100, 101-400, >400)^8^ or four (Agatston scores 0, 1-100, 101-300, >300) risk categories are typically used.^2^ Previous studies investigating CAC scoring in CSCT^4^,^14^ defined four risk groups (0, 1-100, 101-400, >400). In this work, categorization of patients in five risk groups was evaluated to allow differentiation between zero and positive score patients, as well as differentiation between low and intermediate CVD risk patients.

Clinically, it is of key importance to distinguish patients without any calcium and those with positive scores. Thirteen patients had a zero calcium score on dedicated CSCT scan. Manual calcium scoring in CTAC scans at rest and stress identified 12 and 13 of those 13 patients, respectively. However, 5 and 7 additional patients were assigned to the very low CVD risk in scans at rest and stress, respectively. Automatic calcium scoring identified 13 and 11 of the 13 zero score patients but assigned 11 and 9 additional patients to the very low CVD risk group in CTAC at rest and stress, respectively. This demonstrates that CTAC images underestimate CVD risk even when expert manual annotations are performed. This might be due to high levels of image noise, cardiac motion artifacts, and especially partial volume effects, but further research is needed to reveal the causes.

Comparison of CAC scores between CTAC and CSCT scans is hampered by differences in image acquisition. Due to low in-plane resolution in CTAC compared with CSCT scans (~1.35 vs ~0.35 mm), different tube voltages (100 vs 120 kVp) and lack of ECG gating on CTAC images, we can hardly expect to obtain the same CAC scores in CTAC and CSCT. The results demonstrated that CAC scores in CTAC images were significantly lower than the CAC scores in CSCT, both when CTAC images were scored manually and automatically. To compensate for differences in image acquisition parameters, a previous study corrected the scores obtained in coronary CT angiography scans by determining a multiplication factor to convert Agatston scores in coronary CT angiography to Agatston scores in CSCT.^15^ However, this factor depends on the data at hand and the assumption that the relationship between the scores is linear. Hence, we compensated for these differences by ranking the CAC scores in CTAC scans, as we can assume that CSCT provides the reference risk category and that ranking of the CVD risk among the patients remains the same regardless of the image acquisition technique.^13^

This study has several limitations. First, calcium scoring in CTAC images was compared with clinically acquired scores in CSCT scans by different observers. In addition, during scoring of CTAC images, CSCT scans were not available for review. This allowed independent scoring but it also likely introduced inter-observer variabilities thereby hampering exclusive evaluation of the automatic scoring. Separate scoring of CTAC and CSCT scans have resulted in a decreased agreement between both manual and automatic scores in CTAC with the scores in CSCT. Second, correspondence between calcifications in all available scans of the same patient was not analyzed. Due to differences in image acquisition, specifically partial volume effect and cardiac motion, this would have been very challenging. Consequently, only patient-based analysis was performed. However, this is in agreement with clinical analysis and previous studies.^4^,^16^,^17^ Third, CSCT scans were scored using scanner vendor software, while CAC scoring in CTAC images was performed using custom software. This might have caused differences in CAC quantification. Nevertheless, we expect that the disagreement caused by CAC quantification software is minor. Fourth, the speed of the automatic scoring algorithm was not at all optimized as it was implemented within the research environment. It is likely that this process can be performed in a much shorter time in the future. Finally, images included in the study were acquired in a single hospital using a scanner from a single vendor. Future studies are needed to evaluate whether the results generalize to multicenter settings.

In conclusion, automatic assignment of CVD risk based on CAC scoring from CTAC images acquired during rest and stress for PET/CT is feasible. Even though the exact score correspondence between automatic CAC scores in CTAC and CSCT scores is limited due to different image acquisition parameters, the automatic method is able to assign CVD risk to patients using CTAC scans with good agreement. This automated approach may allow routine cardiovascular risk assessment from the CTAC component of PET/CT without any additional radiation dose or scan time.

## New Knowledge Gained

The evaluated machine learning method for automatic calcium scoring can be used to determine CVD risk in CTAC images.

## Electronic supplementary material

Below is the link to the electronic supplementary material.

Supplementary material 1 (PPTX 597 kb)

## Abbreviations

CAC: Coronary artery calcium
CAD: Coronary artery disease
CSCT: Calcium scoring CT
CT: Computed tomography
CTAC: CT attenuation correction
CVD: Cardiovascular disease
CVE: Cardiovascular events
HU: Hounsfield unit
MPI: Myocardial perfusion imaging
PET: Positron emission tomography
SPECT: Single-photon emission computed tomography
