# Supplementary material for: Automatic determination of cardiovascular risk by CT attenuation correction maps in Rb-82 PET/CT
Source: J Nucl Cardiol. 2017 Apr 4;25(6):2133–42. doi: 10.1007/s12350-017-0866-3 (PMC5628109; doi:10.1007/s12350-017-0866-3)
Supplement: Supplementary file 1 — Supplementary material 1 (PPTX 597 kb) [file 12350_2017_866_MOESM1_ESM.pptx]

## Slide 1
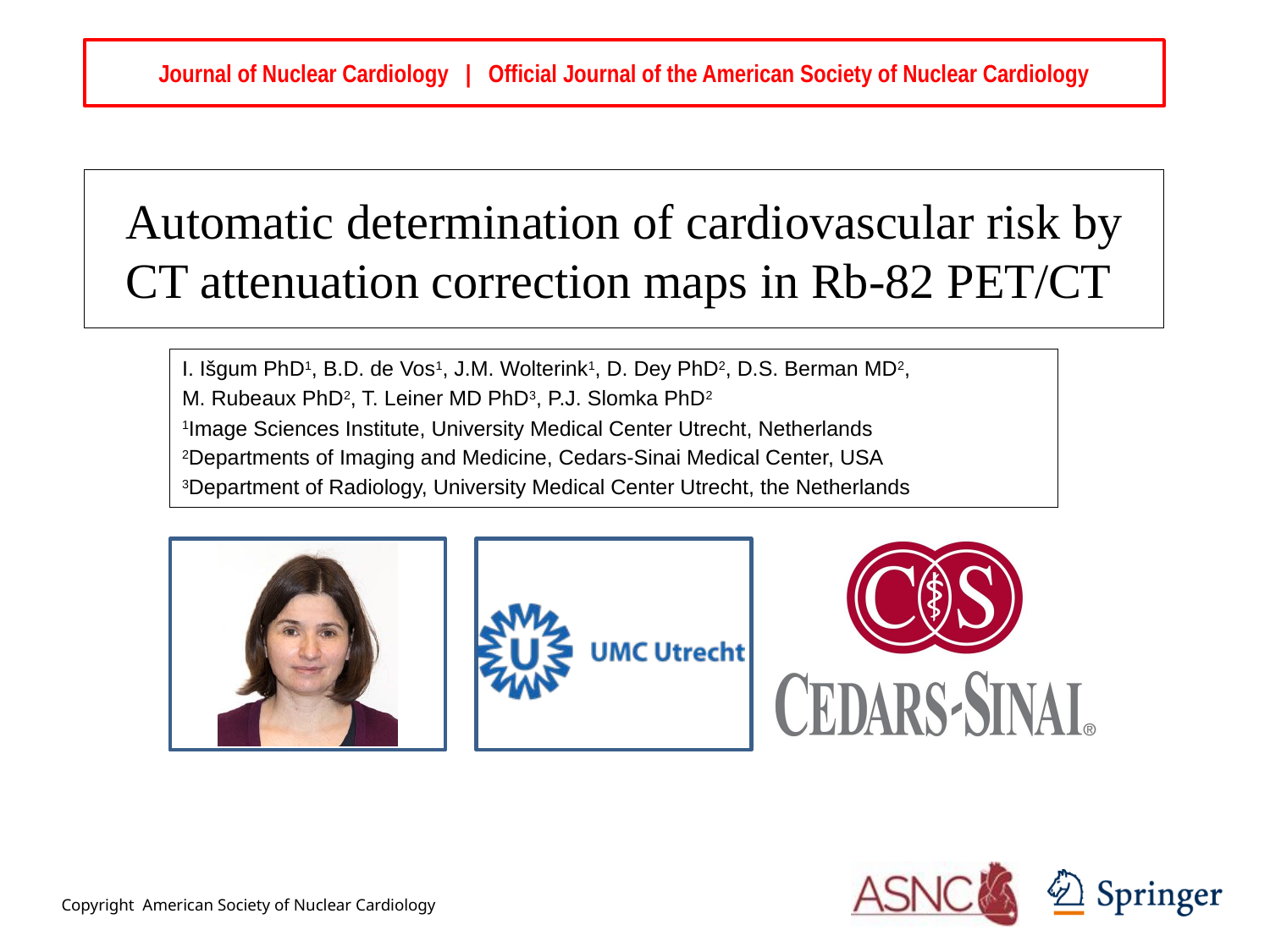

Journal of Nuclear Cardiology | Official Journal of the American Society of Nuclear Cardiology
# Automatic determination of cardiovascular risk by CT attenuation correction maps in Rb-82 PET/CT
I. Išgum PhD1, B.D. de Vos1, J.M. Wolterink1, D. Dey PhD2, D.S. Berman MD2,
M. Rubeaux PhD2, T. Leiner MD PhD3, P.J. Slomka PhD2
1Image Sciences Institute, University Medical Center Utrecht, Netherlands
2Departments of Imaging and Medicine, Cedars-Sinai Medical Center, USA
3Department of Radiology, University Medical Center Utrecht, the Netherlands
Head shot of author
required
Copyright American Society of Nuclear Cardiology

## Slide 2
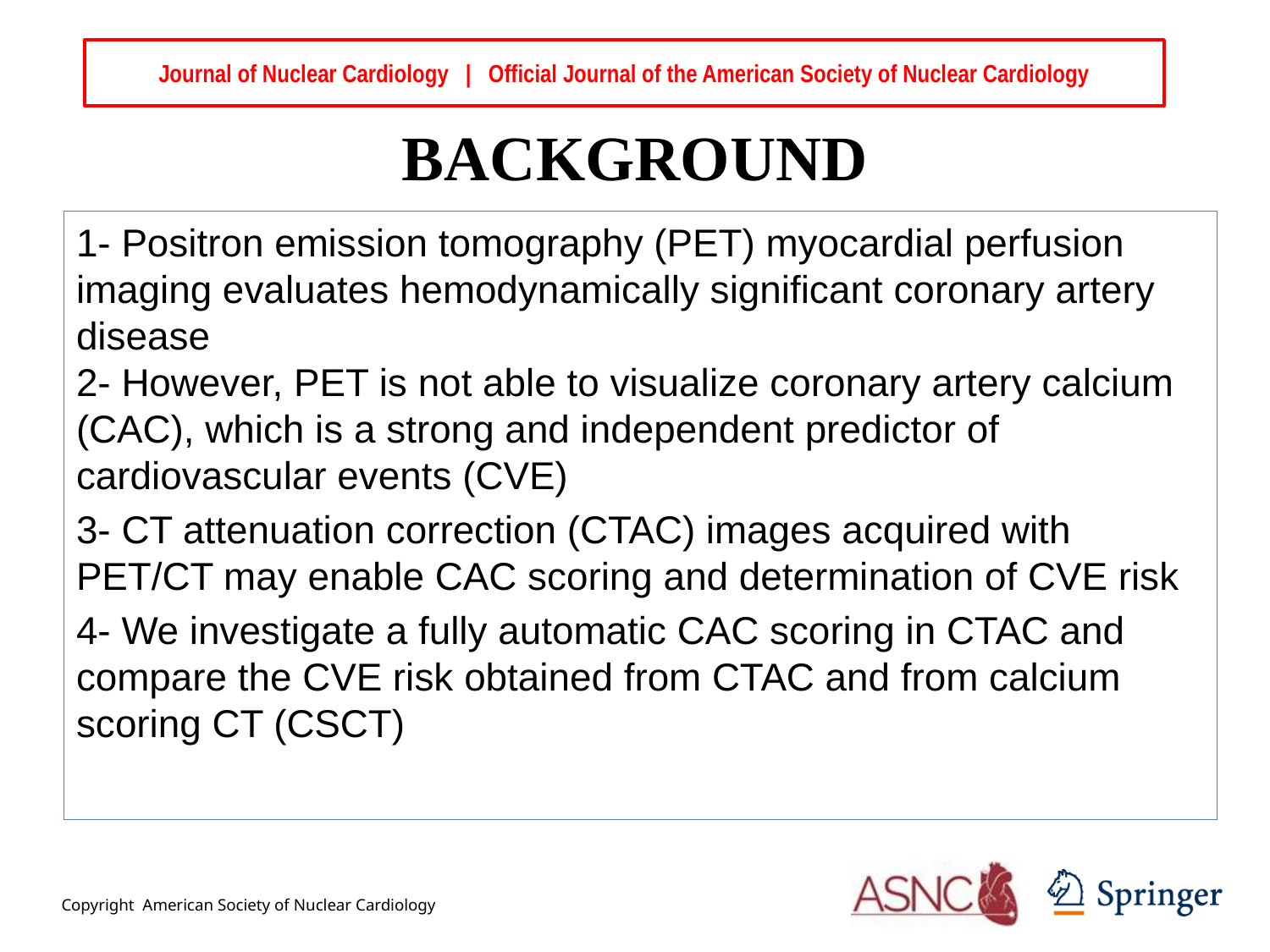

Journal of Nuclear Cardiology | Official Journal of the American Society of Nuclear Cardiology
# BACKGROUND
1- Positron emission tomography (PET) myocardial perfusion imaging evaluates hemodynamically significant coronary artery disease
2- However, PET is not able to visualize coronary artery calcium (CAC), which is a strong and independent predictor of cardiovascular events (CVE)
3- CT attenuation correction (CTAC) images acquired with PET/CT may enable CAC scoring and determination of CVE risk
4- We investigate a fully automatic CAC scoring in CTAC and compare the CVE risk obtained from CTAC and from calcium scoring CT (CSCT)
Copyright American Society of Nuclear Cardiology

## Slide 3
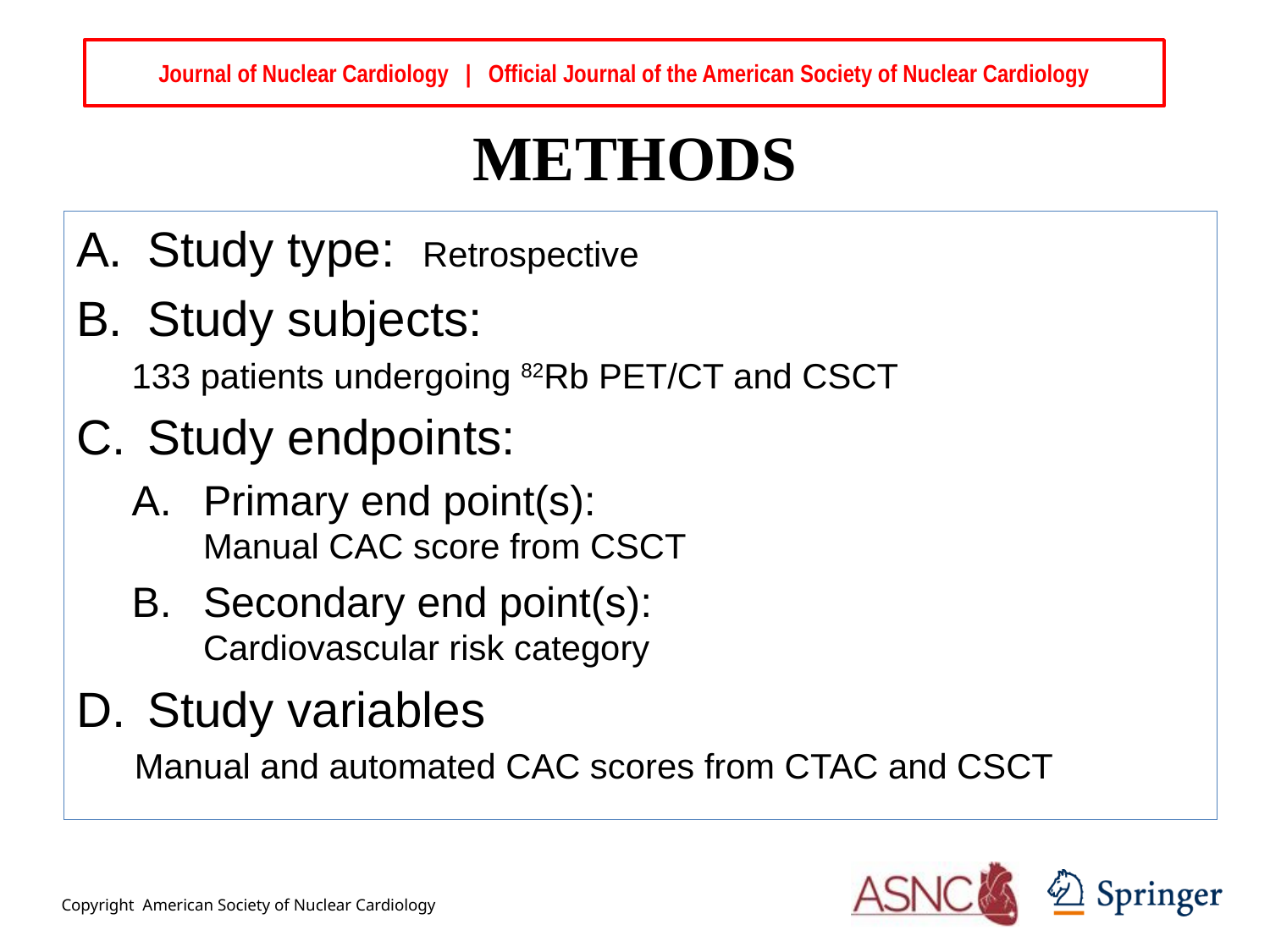

Journal of Nuclear Cardiology | Official Journal of the American Society of Nuclear Cardiology
# METHODS
Study type: Retrospective
Study subjects:
133 patients undergoing 82Rb PET/CT and CSCT
Study endpoints:
Primary end point(s):
Manual CAC score from CSCT
Secondary end point(s):
Cardiovascular risk category
Study variables
 Manual and automated CAC scores from CTAC and CSCT
Copyright American Society of Nuclear Cardiology

## Slide 4
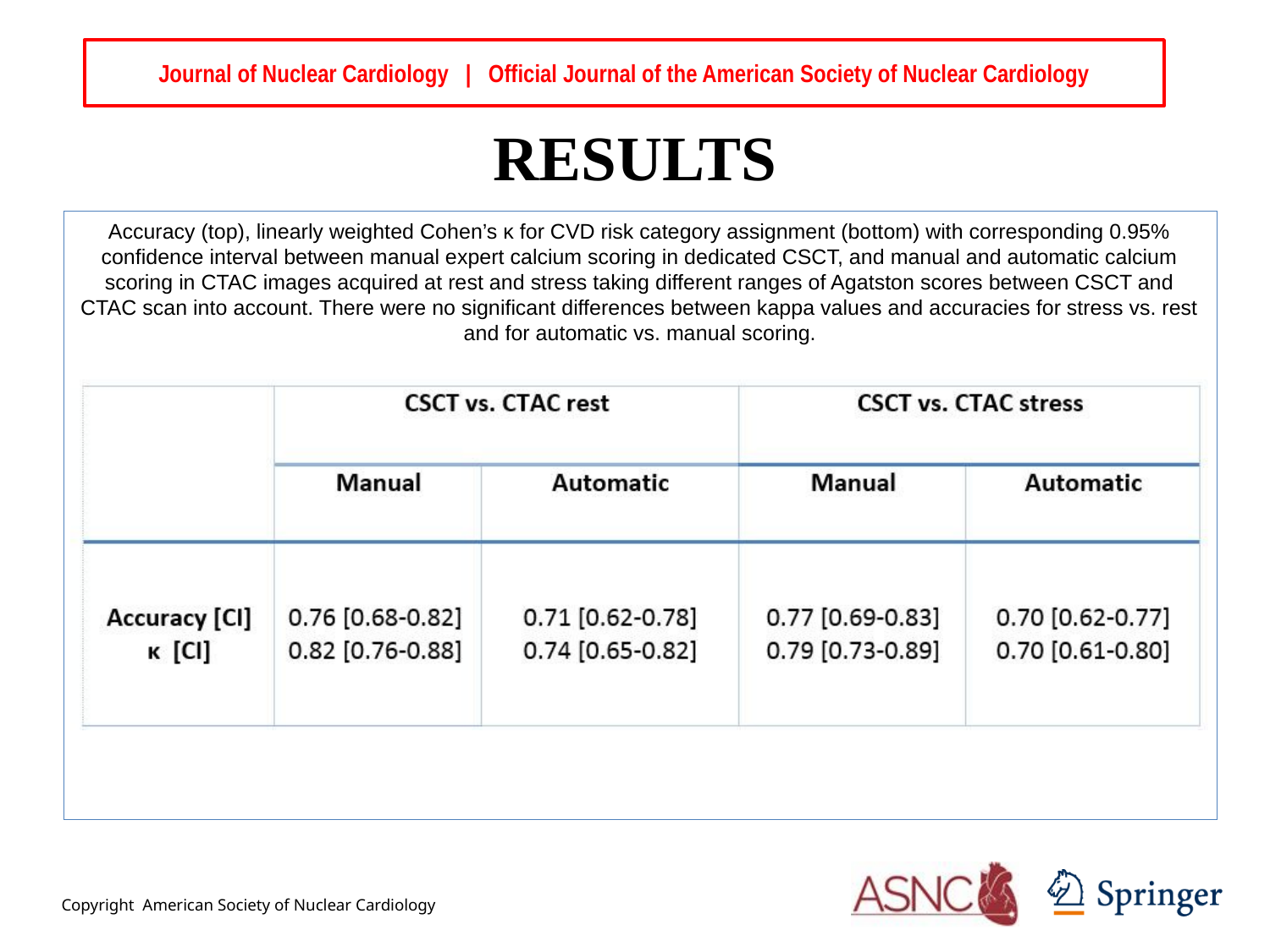

Journal of Nuclear Cardiology | Official Journal of the American Society of Nuclear Cardiology
# RESULTS
Accuracy (top), linearly weighted Cohen’s κ for CVD risk category assignment (bottom) with corresponding 0.95% confidence interval between manual expert calcium scoring in dedicated CSCT, and manual and automatic calcium scoring in CTAC images acquired at rest and stress taking different ranges of Agatston scores between CSCT and CTAC scan into account. There were no significant differences between kappa values and accuracies for stress vs. rest and for automatic vs. manual scoring.
Copyright American Society of Nuclear Cardiology

## Slide 5
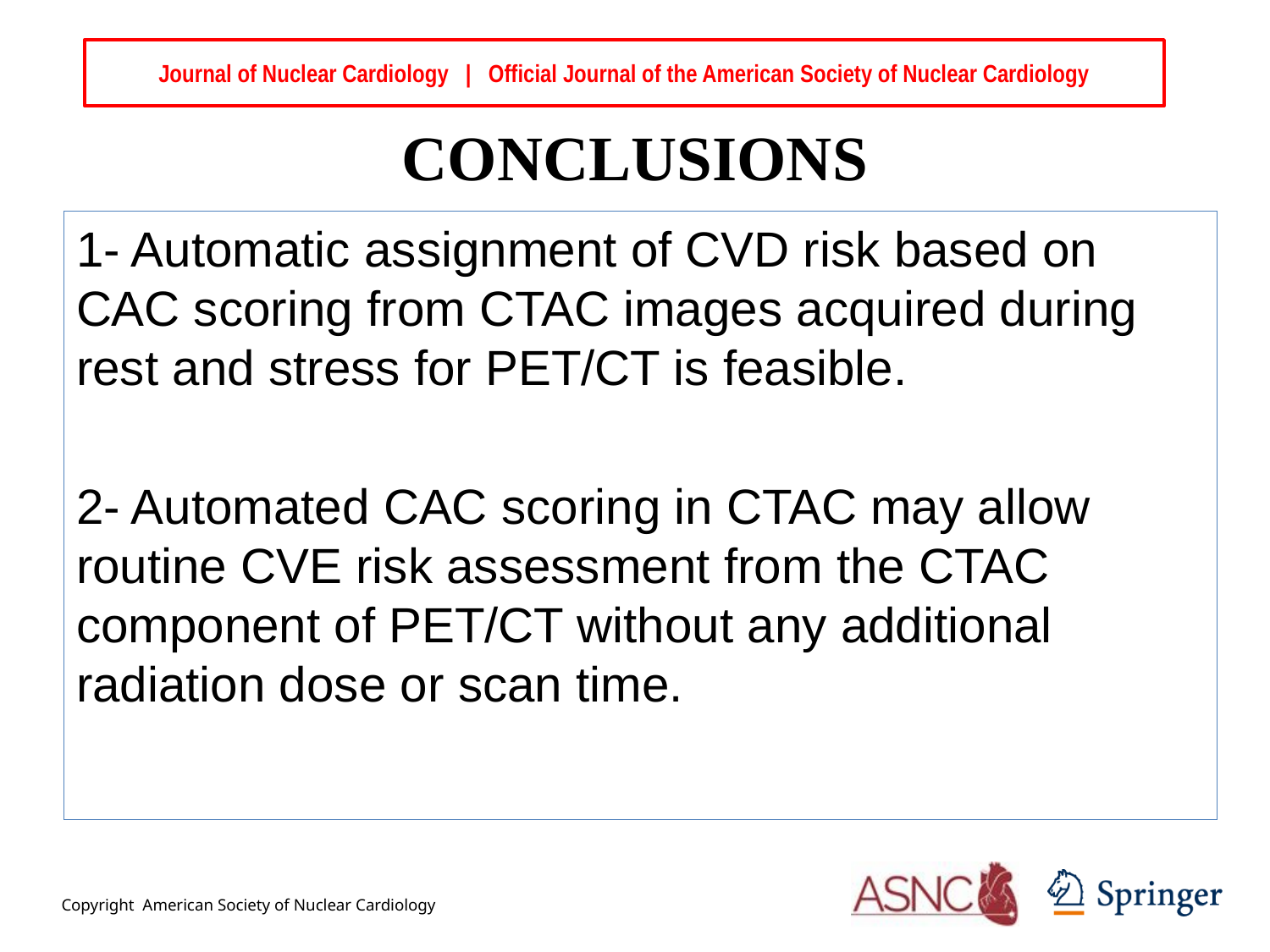

Journal of Nuclear Cardiology | Official Journal of the American Society of Nuclear Cardiology
# CONCLUSIONS
1- Automatic assignment of CVD risk based on CAC scoring from CTAC images acquired during rest and stress for PET/CT is feasible.
2- Automated CAC scoring in CTAC may allow routine CVE risk assessment from the CTAC component of PET/CT without any additional radiation dose or scan time.
Copyright American Society of Nuclear Cardiology
